# Reproductive outcomes among Canadian-born and immigrant women with infertility in Ontario: a population-based cohort study

**DOI:** 10.1093/humrep/deag144

**Published:** 2026-09-24

**Authors:** A Mishra, M Velez, S Yang, N Dayan

**Affiliations:** Department of Epidemiology, Biostatistics and Occupational Health, McGill University, Montreal, QC, Canada; Research Institute, McGill University Health Centre, Montreal, QC, Canada; Research Institute, McGill University Health Centre, Montreal, QC, Canada; Department of Obstetrics and Gynecology, McGill University, Montreal, QC, Canada; ICES, Toronto, ON, Canada; Department of Epidemiology, Biostatistics and Occupational Health, McGill University, Montreal, QC, Canada; Department of Epidemiology, Biostatistics and Occupational Health, McGill University, Montreal, QC, Canada; Research Institute, McGill University Health Centre, Montreal, QC, Canada

**Keywords:** infertility, pregnancy, live birth, immigrants, Canada

## Abstract

**STUDY QUESTION:**

Do reproductive outcomes, including live birth rates, differ between Canadian-born women and immigrant women with infertility in Ontario, Canada?

**SUMMARY ANSWER:**

Immigrant women with infertility, particularly refugees, had lower probabilities of live birth within 2 years of diagnosis compared with Canadian-born counterparts.

**WHAT IS KNOWN ALREADY:**

Immigrant populations experience barriers to fertility care, yet population-level data on reproductive outcomes among immigrant women diagnosed with infertility are limited.

**STUDY DESIGN, SIZE, DURATION:**

This population-based retrospective cohort study used linked administrative and registry data from ICES (formerly the Institute for Clinical Evaluative Sciences) Ontario between 2010 and 2019, with follow-up through 2021. The cohort comprised 254 463 women aged 18–55 years with a new physician-diagnosed infertility visit (OHIP 628), including 177 289 Canadian-born (69.7%) women, 66 131 non-refugee immigrants (26.0%), and 11 043 refugee immigrants (4.3%).

**PARTICIPANTS/MATERIALS, SETTING, METHODS:**

Immigration status (Canadian-born, non-refugee immigrant, refugee immigrant) was derived from Immigration, Refugees and Citizenship Canada (IRCC-PR) data. The primary outcome was live birth within 2 years of infertility diagnosis. Secondary outcomes included time-to-pregnancy and birth by assisted conception. Relative risks were estimated using log-binomial regression and fecundability odds ratios (FORs) using discrete-time Cox models, adjusting for age, parity, income quintile, rurality, chronic conditions, and index year. Analyses followed the STROBE reporting guidelines.

**MAIN RESULTS AND THE ROLE OF CHANCE:**

Among 177 289 Canadian-born, 66 131 non-refugee immigrant, and 11 043 refugee immigrant women, live birth within 2 years occurred in 68 394 (38.5%) Canadian-born women, 22 363 (33.8%) non-refugees, and 3525 (31.9%) refugees, with aRRs of 0.96 (95% CI 0.94–0.97) and 0.90 (95% CI 0.87–0.94), respectively, compared to the Canadian-born cohort. The median time-to-pregnancy was 10 months (IQR 4–23) for Canadian-born women and 11 months (IQR 4–24) for immigrants, with reduced fecundability among both non-refugee (aFOR 0.97, 95% CI 0.96–0.99) and refugee immigrants (aFOR 0.89, 95% CI 0.86–0.91). Among those who achieved pregnancy, births by assisted conception occurred in 21 449 (24.5%) Canadian-born women, 5174 (17.6%) non-refugees, and 666 (14.4%) refugees, with adjusted RRs of 0.73 (95% CI 0.70–0.75) and 0.64 (95% CI 0.60–0.69), respectively, compared to the Canadian-born cohort.

**LIMITATIONS, REASONS FOR CAUTION:**

Administrative data capture physician-diagnosed infertility, which may under-represent immigrants who are less likely to receive a diagnosis, leading to potential selection bias and underestimation of disparities. Residual confounding and incomplete ascertainment of conception type or social determinants remain possible.

**WIDER IMPLICATIONS OF THE FINDINGS:**

Despite universal health coverage and a publicly funded IVF program, inequities in fertility outcomes persist for immigrants, especially refugees. These findings underscore the need for equity-focused fertility policy that addresses cultural acceptability, diagnostic access, and integration of mental-health supports in reproductive care.

**FUNDING:**

This study was supported by the Canadian Institutes of Health Research (CIHR) Project Grant *Impact of Infertility and its Treatment on Women’s Mental Health* (FRN 165840). The research was conducted at ICES, which is funded by an annual grant from the Ontario Ministry of Health and the Ministry of Long-Term Care.

**DISCLOSURES:**

None of the authors report conflicts of interest relevant to this study.

**TRIAL REGISTRATION NUMBER:**

Not applicable.

## Introduction

Infertility, defined as the inability to conceive after 12 months of unprotected intercourse, affects ∼12–16% of Canadian couples (Bushnik *et al.*, 2012; Canadian Fertility and Andrology Society, 2002; Chambers *et al.*, 2009). In the more developed countries in Europe and the UK, 12-month infertility prevalence estimates range from roughly 3.5–16.7% (Boivin *et al.*, 2007) and around one in seven couples in the UK are affected (Datta *et al.*, 2016). These figures reflect substantial personal and system-level costs, including treatment expenses, travel and missed work, and rising demand for specialized reproductive care.

To improve access to ARTs, Ontario introduced the publicly funded Ontario Fertility Program (OFP) in 2015, which covers one IVF cycle per eligible individual (Assal *et al.*, 2019). The program was intended to reduce financial barriers and increase equitable access to fertility care across sociodemographic groups. (Cantor *et al.*, 2023; Winsor *et al.*, 2020).

However, disparities in treatment utilization and outcomes may remain, particularly among immigrant populations. Immigrant women are not a homogeneous population; migration pathway, country or region of origin, socioeconomic position, education, language proficiency, and reasons for migration may shape healthcare access differently across immigrant groups. For example, women who migrate voluntarily from high-income countries may have different social and economic resources than refugee women, who may be more likely to experience forced displacement, interrupted care, trauma exposure, and socioeconomic instability (De Maio, 2010; Khanlou *et al.*, 2017; Mohammadi *et al.*, 2024) (Supplementary Table S1). Immigrant women, particularly recent arrivals and those from under-resourced regions, may experience compounding barriers to infertility care, including language barriers, limited health literacy, cultural stigma, and systemic discrimination (Gill *et al.*, 2019; Zelkowitz *et al.*, 2015).

In particular, little is known about whether reproductive outcomes differ by immigrant status, refugee classification, or recency of immigration among women with diagnosed infertility (Winsor *et al.*, 2020). Despite universal health coverage in Canada, access to infertility care may not be equitable across populations (World Health Organisation Guideline Development Group for Infertility *et al.*, 2026). Immigrant women may face barriers to timely diagnosis and treatment, including language challenges, limited familiarity with the healthcare system, cultural stigma surrounding infertility, and difficulties navigating specialist referral pathways (Gill *et al.*, 2019; Khanlou *et al.*, 2017; Kirmayer *et al.*, 2011). These barriers may delay diagnosis, reduce uptake of ARTs, or limit continuity of care, ultimately affecting reproductive outcomes (Gill *et al.*, 2019; Zelkowitz *et al.*, 2015). Refugee women may experience additional structural vulnerabilities, including socioeconomic instability, trauma exposure, and disrupted healthcare prior to arrival, which may further impede access to infertility services (De Maio, 2010; Wanigaratne *et al.*, 2018).

This study examines whether reproductive outcomes, including live birth rates, and, among those who do become pregnant, birth by assisted conception and time-to-pregnancy, differ by immigrant status, distinguishing between non-refugee and refugee immigrants, compared with Canadian-born women within a population of women with infertility in Ontario. The aim was to assess how inequities in access to infertility diagnosis, treatment uptake, and continuity of care may contribute to these differences.

## Materials and methods

### Ethics approval

Research ethics board (REB) approval was obtained from both the McGill University Health Centre (MUHC) (Nagano identifier—2020-6294) and Queen’s University Health Sciences (TRAQ 6028243).

### Data sources and study population

We conducted a population-based retrospective cohort study using linked provincial health administrative databases at ICES (formerly the Institute for Clinical Evaluative Sciences), Ontario, adhering to the Strengthening the Reporting of Observational Studies in Epidemiology (STROBE) guidelines (von Elm *et al.*, 2007).

ICES is a not-for-profit research institute and a prescribed entity under Ontario’s privacy legislation, capturing records for ∼13 million Ontarians eligible for universal health coverage since 1986 (www.ices.on.ca) (ICES, 2025). All records are securely linked using unique encrypted identifiers (Canadian Institute for Health Information, 2025; ICES, 2025). The databases used were: the Ontario Health Insurance Plan (OHIP) to capture infertility diagnoses, physician claims, and outpatient mental health service visits; Discharge Abstract Database (DAD) to capture inpatient hospital records, obstetrical outcomes, and maternal comorbidities; National Ambulatory Care Reporting System (NACRS) for emergency department visits and hospital- and community-based ambulatory care encounters relevant to mental health; Ontario Mental Health Reporting System (OMHRS) for psychiatric admissions and mental health diagnoses; and Registered Persons Database (RPDB) to identify demographic information such as age, sex, neighbourhood income quintile, and rurality; the Immigration, Refugees and Citizenship Canada—Permanent Residents (IRCC-PR) database to obtain immigration status and years since landing, with individuals categorized as non-refugee immigrants or refugees, with the latter identified using ‘landing category codes’ designating government-assisted, privately sponsored or protected-person entry classes (Chiu *et al.*, 2016); and the Better Outcomes Registry & Network (BORN/NIDAY) to capture pregnancy outcomes, conception type, gestational age, and delivery records (Supplementary Table S2).

Previous validation studies have demonstrated the high quality of these linked data sources for perinatal outcomes. In particular, BORN Ontario captures more than 99% of hospital births in the province and has been shown to provide valid information on gestational age, live birth status, and infertility treatment records (Dayan *et al.*, 2019, 2022). Although BORN does not capture home births, these represent a very small proportion of deliveries in Ontario; Statistics Canada reported 3187 non-hospital births in Ontario in 2019 and 3861 in 2020, corresponding to ∼2.2–2.8% of annual Ontario births using BORN Ontario annual birth numbers (Statistics Canada, 2019; Association of Ontario Midwives, 2022), and prior work indicates their exclusion has minimal impact on outcome estimates (Dayan *et al.*, 2019; Dunn *et al.*, 2019).

We included women aged 18–55 years who had at least one documented infertility diagnosis (OHIP code 628) between 1 January 2010 and 31 December 2019. Individuals were included at the time of their first recorded infertility diagnosis (index date), resulting in one record per individual in the analytic dataset. The use of OHIP 628 to identify infertility visits has been validated in Ontario administrative data, demonstrating >90% positive predictive value and strong agreement with chart-confirmed clinical assessments (Rae *et al.*, 2023), and is widely used in population-based fertility research (Dayan *et al.*, 2022; Declercq *et al.*, 2014; Fine *et al.*; Fine *et al.*, 2022; Velez *et al.*, 2023).

Individuals with invalid or missing ICES identifiers, implausible demographic data, or fewer than 2 years of OHIP eligibility prior to the index infertility diagnosis were excluded (n = 42 765; 7.8% of 548 339) to ensure a consistent 2-year lookback period for ascertainment of prior infertility diagnoses and baseline characteristics. Those with a documented infertility-related diagnosis or treatment in the 2 years before their first OHIP 628 claim (n = 42 463; 7.7%) were excluded to ensure a new index consultation (using a 2-year lookback to 2008); individuals not residing in Ontario at the time of diagnosis (n = 1165; 0.2%) and those with missing immigration status (n = 1698; 0.3%) were also excluded to ensure accurate exposure classification and complete follow-up (Fig. 1; Supplementary Table S3).

**Figure 1.**
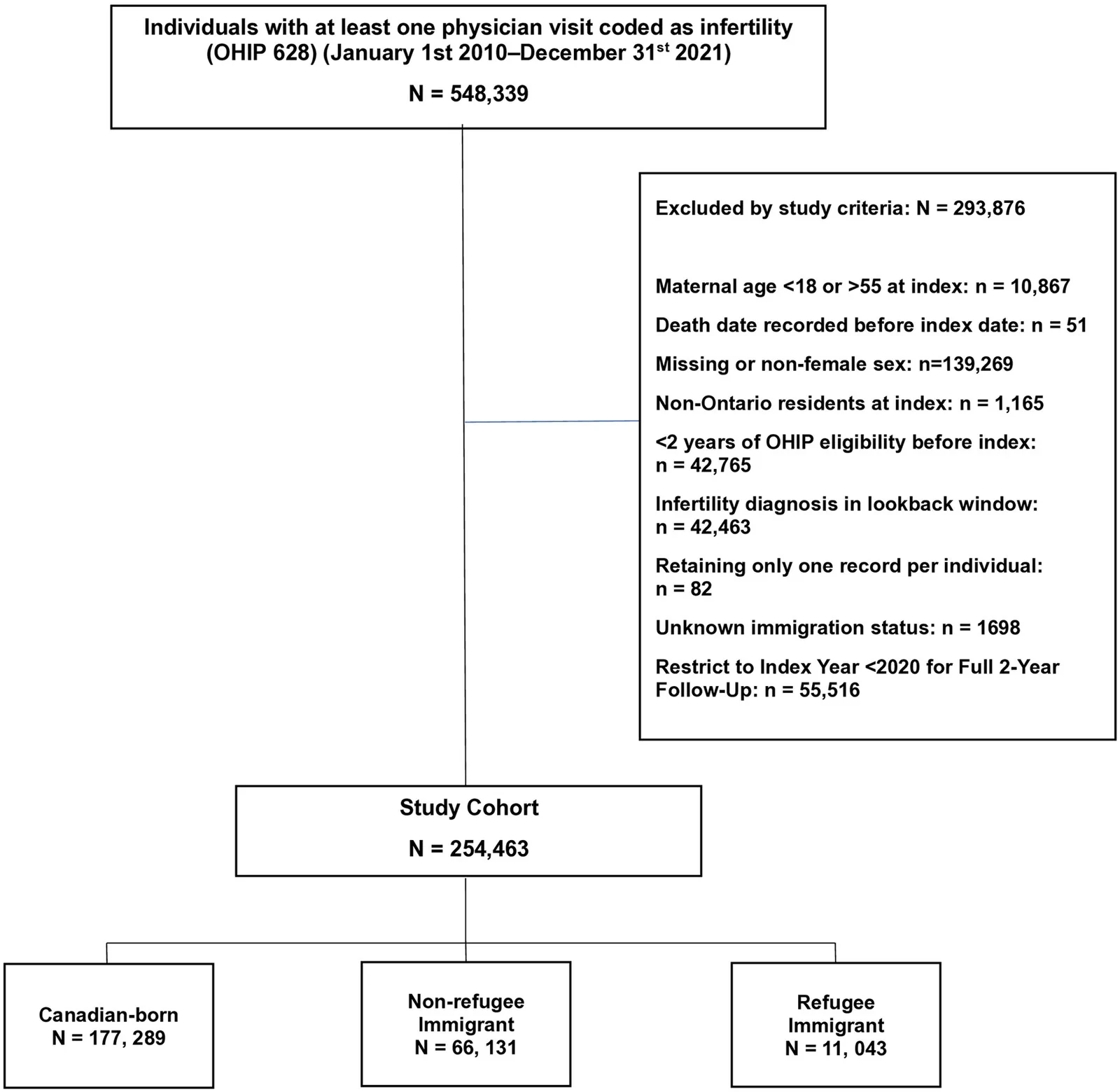
Study flow diagram.

### Exposure and outcome variables

The primary exposure of immigrant status was categorized as refugee immigrant and non-refugee immigrant, while Canadian-born women served as the referent.

The primary outcome was incident live birth within 2 years of the index infertility diagnosis, identified using BORN/NIDAY records. Live birth was defined as the delivery of a liveborn infant at 23 weeks’ gestation or later, in accordance with viability thresholds used in perinatal research (Public Health Agency of Canada, 2020; Varner *et al.*, 2020).

Among individuals who achieved a pregnancy, we assessed additional outcomes birth by assisted conception (i.e. use of invasive ARTs such as IVF and ICSI, and non-invasive approaches such as ovulation induction [OI] and IUI), and time to pregnancy, defined as the interval between the index visit for code OHIP 628 and the estimated conception date (delivery date minus gestational age at birth) (Ishikawa *et al.*, 2018), both derived from BORN Ontario (Supplementary Table S4).

Each individual contributed only once to the live-birth analysis, which assessed whether any qualifying live birth occurred within 2 years of the index infertility diagnosis. For pregnancy-level secondary analyses, only the first recorded qualifying pregnancy per individual was retained; multiple gestations were treated as a single pregnancy event.

### Covariates

Covariates included factors with the potential to confound the association between immigration status and reproductive outcomes. These included: age at index infertility diagnosis (continuous) (Cantor *et al.*, 2023); parity (nulliparous vs parous) (Varner *et al.*, 2020); ‘neighbourhood income quintile’ (1 [lowest] to 5 [highest], with quintile 1 as the reference) (Varner *et al.*, 2020); rurality (yes vs no) (Jaworsky *et al.*, 2020); and chronic health conditions (Liang *et al.*, 2024), including HIV (Yudin *et al.*, 2010), hypertension (Smith *et al.*, 2021), diabetes (Mattsson *et al.*, 2021), asthma (Crowe *et al.*, 2020), obesity (Bhattacharya S *et al.*, 2009), congestive heart failure (Smith *et al.*, 2021), chronic obstructive pulmonary disease (Liang *et al.*, 2024), autoimmune disorders (Carp *et al.*, 2012), endocrine disorders (Bendarska-Czerwińska *et al.*, 2022), and thrombosis or thrombophilia (any vs none) (Fatini C *et al.*, 2012) (Supplementary Table S5).

Mental health conditions, including mood, anxiety, psychotic, and substance-related disorders were identified through OMHRS, DAD, NACRS, and OHIP data, based on ≥1 hospitalization or ≥2 outpatient visits within the 2 years before index. This definition captures mental health conditions documented within Ontario healthcare records and may not reflect untreated conditions or diagnoses that occurred prior to arrival in Canada.

### Statistical analysis

Baseline characteristics were summarized using means with SDs for continuous variables and frequencies with percentages for categorical variables, stratified by immigrant status (Canadian-born, non-refugee immigrant, refugee immigrant).

For the primary analyses, log-binomial regression models were used to estimate unadjusted and adjusted relative risks (aRRs) for achieving a live birth within 2 years of the initial consultation for infertility. All models compared non-refugee and refugee immigrants with Canadian-born women (the referent).

Among individuals with a recorded pregnancy, we assessed birth by assisted conception, defined as conception resulting from IVF, ICSI, OI or IUI, compared with natural conception. Because conception type is only captured in BORN Ontario for pregnancies resulting in a recorded delivery, this outcome does not capture ART attempts, failed ART cycles, or pregnancies ending before 20 weeks’ gestation. Discrete-time Cox proportional hazards models were used to examine time to pregnancy, expressed as adjusted fecundability odds ratios (FORs) along with 95% CIs. A FOR < 1 indicates reduced fecundability (lower monthly probability of conception) and FOR > 1 indicates increased fecundability (Loy *et al.*, 2021). Women were followed from the index date until conception and were censored at loss of OHIP eligibility, death, or the administrative end of follow-up (31 December 2023). The upper age limit of 55 years was applied at cohort entry and was not used as a censoring criterion. To assess covariate contributions to attenuation of crude estimates, we conducted stepwise models adding each adjustment domain (sociodemographic, clinical, and reproductive) sequentially.

We conducted additional analyses comparing outcomes before and after implementation of the Ontario Fertility Program (2010–2014 vs 2015–2019), by recency of immigration (<5 vs ≥5 years since arrival), and descriptively summarized world region of origin across immigration categories. As a sensitivity analysis, we repeated the primary and key secondary analyses after excluding immigrants who had arrived in Canada within 5 years of the index infertility diagnosis, while retaining Canadian-born women as the reference group. This analysis assessed whether findings were robust to potential under-ascertainment of health conditions and healthcare encounters among recent arrivals.

We performed complete case analysis for variables with <5% missingness. Gestational age at birth was obtained primarily from the BORN Ontario registry, which integrates information from prenatal screening, antenatal visits, and delivery records. Among cohort records, gestational age was nearly complete, with only 0.13% of records in the legacy NIDAY perinatal database and 0.002% in the Birth Information System (BIS) missing this variable. Given the negligible level of missingness, these few records were excluded rather than imputed (Margulis *et al.*, 2023).

Our study was designed assuming a baseline live birth rate of 30% among infertile women (Canadian Fertility and Andrology Society, 2002; Smith *et al.*, 2015). Given the available sample sizes, the study had >90% power (two-sided α = 0.05) to detect a minimum absolute difference in live birth of 0.65 percentage points for non-refugee immigrants versus Canadian-born women and 1.39 percentage points for refugee immigrants versus Canadian-born women. Live birth within 2 years was specified as the primary outcome and interpreted using a two-sided α  =  0.05. Birth by assisted conception and time-to-pregnancy were considered key secondary outcomes, while Ontario Fertility Program era-stratified analyses and years-since-landing analyses were considered exploratory. To account for multiple comparisons across secondary and exploratory analyses, we applied the Benjamini–Hochberg false discovery rate procedure across 19 retained comparisons, using a target FDR threshold of *q* < 0.05.

All statistical analyses were performed using SAS version 9.4 (SAS Institute Inc., Cary, NC, USA) via SAS Enterprise Guide within the ICES Research Analytic Environment; statistical significance was set at alpha = 0.05.

## Results

We identified 254 463 individuals with infertility in Ontario between 2010 and 2019: 177 289 (69.7%) Canadian-born, 66 131 (26.0%) non-refugee immigrants, and 11 043 (4.3%) refugees (Table 1). Among the 254 463 women in the infertility cohort, 94 282 experienced a live birth within 2 years of their index infertility diagnosis. Pregnancy-level secondary analyses were based on the first recorded qualifying pregnancy per individual. Canadian-born women were slightly younger at the index infertility consultation (mean age 33.3 years [SD 6.4]) than non-refugee immigrants (34.7 years [SD 6.1]; mean difference 1.4 years) and refugee immigrants (34.0 years [SD 6.9]; mean difference 0.7 years). Canadian-born women were also more likely to reside rurally, while refugees were more concentrated in the lowest income quintile. Chronic conditions were most common among Canadian-born, and mental health diagnoses were more prevalent among Canadian-born women and refugees compared with non-refugee immigrants (Table 1, Supplementary Table S6).

**Table 1.** Characteristics of N = 254 463 individuals with diagnosed infertility in Ontario between 2010 and 2019 by immigration status.

Characteristic	Canadian-born (N = 177 289)	Non-refugee immigrant (N = 66 131)	Refugee immigrant (N = 11 043)	Standardized differences 1^†^	Standardized differences 2^†^
Maternal age at initial infertility consultation (years)					
Mean (SD)	33.3 (6.4)	34.7 (6.1)	34.0 (6.9)	0.23	0.11
18–24	12023 (6.8)	1859 (2.8)	804 (7.3)	0.17	0.02
25–29	37878 (21.4)	11265 (17.0)	2324 (21.0)	0.11	0.01
30–34	57735 (32.6)	21196 (32.1)	2944 (26.7)	0.01	0.13
35–39	42678 (24.1)	18237 (27.6)	2588 (23.4)	0.08	0.01
40–44	18067 (10.2)	9454 (14.3)	1607 (14.6)	0.13	0.14
≥45	7331 (4.1)	3246 (4.9)	613 (5.6)	0.04	0.07
Rural residence	11166 (6.3)	501 (0.8)	41 (0.4)	0.30	0.33
Neighborhood income quintile					
1 (lowest)	29032 (16.4)	16751 (25.3)	4413 (40.0)	0.22	0.54
2	34237 (19.3)	14564 (22.0)	2350 (21.3)	0.07	0.05
3	37317 (21.0)	13558 (20.5)	1855 (16.8)	0.01	0.11
4	39910 (22.5)	12468 (18.9)	1542 (14.0)	0.09	0.22
5 (highest)	36793 (20.8)	8790 (13.3)	883 (8.0)	0.20	0.37
Nulliparity	158142 (89.2)	58492 (88.4)	9411 (85.2)	0.02	0.20
Medical history					
Any chronic health condition^║^	51196 (28.9)	10443 (15.8)	2222 (20.1)	0.32	0.20
Mental illness^‡‡^	32982 (18.6)	7066 (10.7)	1704 (15.4)	0.23	0.08
Physician at first infertility consult^¶^					
Family physician	115519 (65.2)	39251 (59.4)	6894 (62.4)	0.12	0.06
OB/GYN	61770 (35.8)	26880 (40.6)	4149 (37.6)	0.12	0.06
Prenatal healthcare Characteristics					
Prenatal visit*					
Initial prenatal visit	1015 (0.6)	125 (0.2)	20 (0.2)	0.02	0.23
Antenatal preventative health assessment	1285 (0.7)	195 (0.3)	32 (0.3)	0.32	0.17
Minor prenatal assessment	74698 (42.1)	26521 (40.1)	4174 (37.8)	0.21	0.11
High-risk prenatal assessment	5979 (3.4)	1929 (2.9)	294 (2.7)	0.08	0.01
No recorded prenatal visit	4664 (2.6)	601 (0.9)	96 (0.9)	0.15	0.30
Prenatal ultrasound*					
Complete ultrasound (on or after 16 weeks gestation)	22334 (12.6)	7306 (11.0)	1296 (11.7)	0.08	0.01
Maternal serum screening program ultrasound (before 16 weeks gestation)	17249 (9.7)	5414 (8.2)	884 (8.0)	0.13	0.14
Limited ultrasound (high-risk pregnancy or complications)	32964 (18.6)	10546 (15.9)	1567 (14.2)	0.03	0.13
No recorded ultrasound	15094 (8.5)	6105 (9.2)	869 (7.9)	0.01	0.13

Data shown as frequency with percentages unless otherwise specified.

* Variables available only among individuals who achieved a pregnancy (N = 121628).

† Standardized differences (SD1: Canadian-born vs Non-refugee immigrant; SD2: Canadian-born vs refugee immigrant).

¶ Physician type at first infertility consultation; OB/GYN category includes records with missing specialty data (data missing in <1%).

║ Chronic health condition: presence of ≥1 of the following conditions: asthma, chronic obstructive pulmonary disease (COPD), diabetes mellitus, hypertension, HIV, congestive heart failure, autoimmune diseases, endocrine disorders, thrombosis, and obesity.

‡‡ Mental illness: presence of ≥1 conditions: mood disorders (e.g. depression, bipolar disorder), schizophrenia and psychotic disorders, substance use disorders, self-harm, personality disorders, and other mental health diagnoses.

Within 2 years after the index infertility diagnosis, of 95 134 pregnancies reaching ≥20 weeks, 94 282 (99.1%) resulted in a live birth (Table 2). Compared with Canadian-born women, non-refugee immigrants were less likely to achieve a live birth (22 363/66 131 [33.8%] vs 68 394/177 289 [38.5%], aRR 0.96, 95% CI 0.94–0.97), with greater reductions observed among refugees (live birth 3525/11 043 [31.9%] vs 68 394/177 289 [38.5%], aRR 0.90, 95% CI 0.87–0.94). Immigrants who achieved a pregnancy were also less likely to have a birth by assisted conception compared with Canadian-born women. Median time to pregnancy was slightly longer among immigrants, and both non-refugee and refugee groups demonstrated lower fecundability relative to Canadian-born women (Table 3).

**Table 2.** Association between immigrant status and reproductive outcomes, Ontario, 2010–2019, N = 254 463.

Outcome	Exposure	n/N (%)	Crude RR (95% CI)	Adjusted* RR (95% CI)
Live birth within 2 years	Canadian-Born	68394/177289 (38.5)	Reference	Reference
	Non-Refugee Immigrant	22363/66131 (33.8)	0.87 (0.86–0.90)	0.96 (0.94–0.97)
	Refugee Immigrant	3525/11043 (31.9)	0.83 (0.80–0.86)	0.90 (0.87–0.94)
Birth by assisted conception^‡^	Canadian-Born	21 449/87641 (24.5)	Reference	Reference
	Non-Refugee Immigrant	5174/29371 (17.6)	0.72 (0.70–0.74)	0.73 (0.70–0.75)
	Refugee Immigrant	666/4616 (14.4)	0.60 (0.55–0.63)	0.64 (0.60–0.69)

Non-refugee and refugee immigrant status each compared with Canadian-born. Data shown as frequency (n/N, %), where n = number with the outcome and N = total number in the exposure group. Crude and adjusted relative risks (RR) or fecundability odds ratios (FOR) are presented with 95% CIs.

* Model adjusted for age, parity, income quintile, rurality, and chronic health conditions.

‡ Assisted conception analyses restricted to individuals with a pregnancy; includes IVF, IVF with ICSI, ovulation induction (OI), and IUI.

**Table 3.** Association between immigrant status and time to first pregnancy, N = 254 463.

Immigration category	Time to pregnancy, median (IQR) [months]^†^	Crude FOR (95% CI)	Adjusted* FOR (95% CI)
Canadian-born (N = 87641)	10 (11–106)	Reference	Reference
<6 months:	29758 (33.9)		
6–12 months:	17689 (20.2)		
>12 months:	40194 (45.9)		
Non-refugee immigrant (N = 29371)	11 (13–115)	0.86 (0.85–0.87)	0.97 (0.96–0.99)
<6 months :	9703 (33.0)		
6–12 months:	5812 (19.8)		
>12 months:	13856 (47.2)		
Refugee immigrant (N = 4616)	11 (15–115)	0.79 (0.78–0.81)	0.89 (0.8–0.91)
<6 months :	1528 (33.1)		
6–12 months:	919 (19.9)		
>12 months:	2169 (47.0)		

Time to pregnancy (TTP) and fecundability odds ratios (FOR) shown for each immigration category, with Canadian-born women as the reference group in discrete-time Cox models.

* Model adjusted for age, parity, income quintile, rurality, and chronic health conditions.

† Time to pregnancy calculated among those achieving pregnancy; FOR, fecundability odds ratio.

In stepwise models, sequential adjustment for chronic conditions and parity produced the largest reduction in crude risk estimates between immigrant and Canadian-born women.

In analyses stratified by policy era, immigrants had lower incidences of live birth than Canadian-born before OFP (2010–2014), while these differences narrowed after OFP implementation (2015–2019). Birth by assisted conception remained consistently lower among immigrants, and fecundability gaps narrowed but persisted, particularly for refugees (Supplementary Table S7).

Finally, more recent immigrants (<5 years in Canada) consistently had higher probabilities of live birth, and higher fecundability, compared with longer-term immigrants (≥5 years) (Supplementary Table S8).

In sensitivity analyses excluding immigrants who arrived within 5 years of the index infertility diagnosis, the associations remained similar in direction to the primary analysis. Compared with Canadian-born women, non-refugee immigrants had lower likelihood of live birth (aRR 0.94, 95% CI 0.93–0.95), lower fecundability (aFOR 0.94, 95% CI 0.93–0.96), and lower likelihood of birth by assisted conception (aRR 0.73, 95% CI 0.71–0.76). Corresponding estimates among refugee immigrants were 0.92 (95% CI 0.89–0.95), 0.90 (95% CI 0.87–0.93) and 0.68 (95% CI 0.63–0.73), respectively (Supplementary Table S9).

Immigrant populations were heterogeneous with respect to world region of origin, with variation in regional representation between non-refugee and refugee groups. Among non-refugee immigrants, the largest proportions originated from the Middle East and North Africa (25.2%), whereas refugee immigrants had greater representation from Sub-Saharan Africa (15.1%) (Supplementary Table S10).

After Benjamini–Hochberg false discovery rate correction across 19 secondary and exploratory comparisons, 17 comparisons remained statistically significant at *q* < 0.05. Post-OFP live birth and post-OFP time-to-pregnancy among non-refugee immigrants did not remain statistically significant after correction and were interpreted cautiously (Supplementary Table S11).

## Discussion

In this population-based cohort of over 250 000 women with infertility in Ontario, we observed modest but consistent disparities in reproductive outcomes by immigration status. Within 2 years of diagnosis, the cumulative incidence of live birth was 4.7 percentage points lower among non-refugee immigrants and 6.6 percentage points lower among refugee immigrants compared with Canadian-born women; these differences were attenuated after adjustment for sociodemographic and clinical factors. Median time to pregnancy was slightly longer among immigrants, and fecundability was lower among immigrant women, particularly refugees. Assisted conception was less common among immigrants, especially refugees. Although several associations were statistically significant, the absolute differences were modest. For instance, median time-to-pregnancy differed by about 1 month, underscoring the importance of interpreting these findings in light of their potential clinical relevance and real-world implications.

Because conception type is only available for pregnancies resulting in a recorded delivery, birth by assisted conception should not be interpreted as overall ART uptake among all women with infertility.

Our findings align with growing evidence that immigrant women may face barriers to reproductive care in Canada. In Toronto, Gill *et al.* (2019) found that immigrant women were less likely than Canadian-born women to access fertility services (Gill *et al.*, 2019), even after accounting for socioeconomic factors. Zelkowitz *et al.* (2015) similarly reported lower ART success among immigrant patients compared with their Canadian-born counterparts (Zelkowitz *et al.*, 2015). Refugees, who often face compounded social, economic, and health vulnerabilities, have also been shown to experience the greatest disparities in maternal and perinatal outcomes in Ontario (Wanigaratne *et al.*, 2018). Unlike these studies, which focused on women actively seeking or receiving fertility treatment, our study followed a population-based cohort of all women with an infertility diagnosis, providing a broader view of reproductive outcomes.

The persistent gaps in outcomes despite implementation of the OFP suggest that publicly funded cycles alone may be insufficient to address inequity in access. Previous evaluations of the OFP reported improved access overall but highlighted persistent sociodemographic disparities in utilization (Cantor *et al.*, 2023; Gotz and Jones, 2017; Winsor *et al.*, 2020). Our findings extend this by showing that not only is utilization lower among immigrants, but outcomes following infertility diagnosis remain suboptimal. These findings suggest that policy change may be required beyond funding alone to improve equity in fertility outcomes. These disparities likely reflect differences in access to infertility care, including delays in diagnosis, lower uptake of ARTs, and barriers to sustained engagement with fertility services, particularly among refugee populations (World Health Organisation Guideline Development Group for Infertility *et al.*, 2026). Equitable access will rely on addressing cultural, informational, and system-level barriers that limit participation and engagement among immigrant populations.

Interestingly, more recent immigrants (<5 years) had higher live birth incidences than longer-term immigrants, contrary to our initial hypothesis. This may reflect the healthy immigrant effect, whereby more physically and reproductively robust individuals are more likely to migrate, and positive selection into fertility-seeking behaviours shortly after arrival (Hamilton *et al.*, 2025; Vang *et al.*, 2017), with health and access advantages diminishing over time (De Maio, 2010). Beyond systemic barriers, cultural acceptability and perceived desirability of ARTs may also influence access and utilization, particularly among certain sub-Saharan African and other ethnocultural groups. Previous qualitative studies have highlighted that cultural beliefs, stigma, and differing norms around conception and fertility-seeking can affect both diagnosis and treatment uptake (Khanlou *et al.*, 2017; Kirmayer *et al.*, 2011). These barriers may contribute to underdiagnosis of infertility among immigrant women, potentially leading to selection bias and an underestimation of the true inequity in fertility outcomes.

These findings should also be interpreted in the context of substantial heterogeneity within immigrant populations. As shown in Supplementary Table S10, immigrants in the cohort originated from diverse world regions, with differences in regional composition between non-refugee and refugee groups. Variation in migration pathways, cultural perceptions of infertility, and familiarity with healthcare systems may influence both care-seeking behaviours and treatment uptake and may partially explain differences in reproductive outcomes observed across groups.

Neighbourhood income quintile may also partly capture structural differences related to immigrant and refugee status. Although models adjusted for income quintile, residual confounding by individual-level socioeconomic status, education, language proficiency, and health system navigation barriers may remain.

Future research should move beyond documenting disparities to identifying mechanisms and testing interventions. Qualitative or mixed-methods studies could explore how cultural norms, stigma, and family or gender dynamics shape fertility care experiences across immigrant subgroups, including among male and female partners (Khanlou *et al.*, 2017). These studies could help identify targeted supports and interventions to improve both access and acceptability of infertility treatments. Linking administrative data with clinical ART registries may help clarify whether disparities reflect differences in access, treatment uptake or response (Luke *et al.*, 2012; Smith *et al.*, 2015).

### Limitations

Our study has several limitations. First, administrative data may not fully capture infertility diagnoses or treatments outside provincial funding streams, potentially underestimating assisted conception use. Differential care-seeking across groups could also lead to under-ascertainment of infertility, though all included individuals had documented physician visits, reducing misclassification risk (Bushnik *et al.*, 2012; ICES, 2025). Secondly, infertility was identified using physician billing claims, and individuals were included based on their first recorded infertility diagnosis, resulting in one record per individual in the analytic dataset. As such, we were not able to restrict the cohort to individuals with repeated infertility-coded visits without reconstructing visit counts from raw claims data. Differential care-seeking across groups may therefore lead to under-ascertainment of infertility, particularly among immigrants and refugees with barriers to accessing care. However, sensitivity analyses excluding immigrants who arrived within 5 years of the index diagnosis yielded findings similar in direction to the primary analysis. The slight attenuation of estimates among refugees in this restricted analysis may suggest that refugee-related disparities are greater among newer arrivals, when settlement-related barriers, unfamiliarity with the healthcare system, language barriers, and incomplete capture of prior health history may be most pronounced. The requirement for at least 2 years of prior OHIP eligibility may also have disproportionately excluded recent immigrants who had not yet accrued 2 years of provincial health insurance eligibility. We were also unable to account for certain clinical details, such as cause of infertility, duration of infertility prior to diagnosis, or partner factors, which may influence outcomes (Canadian Fertility and Andrology Society, 2002). BMI data were only available for a subset of women who achieved a pregnancy, and the obesity variable derived from physician billing records may not accurately reflect true prevalence (Rittenberg *et al.*, 2011). Residual confounding by unmeasured social factors, such as education, employment, language proficiency or discrimination, may contribute to the observed associations (Khanlou *et al.*, 2017). Secondary outcomes, including birth by assisted conception and time-to-pregnancy, were ascertainable only among pregnancies resulting in a recorded delivery. Consequently, failed ART cycles and early pregnancy losses before 20 weeks were not captured, which may attenuate observed disparities if these outcomes differ by immigration status. Furthermore, the scope of our measures does not capture the cultural context of fertility decision-making or the acceptability of assisted conception across groups. These unmeasured factors likely contribute to the observed inequities and could explain part of the selection bias if fewer immigrants seek or receive an infertility diagnosis due to cultural or social factors. Mental health conditions were identified using established administrative-data algorithms based on documented healthcare encounters in Ontario. For immigrants and refugees, these data are only available after arrival in Canada and do not capture diagnoses, treatment or symptoms that occurred before immigration. They may also miss untreated conditions, milder symptoms not resulting in healthcare contact, or mental health needs not documented because of stigma, language barriers, or limited access to care. As such, mental health ascertainment may differ by immigration status, limiting interpretation of mental health as an effect modifier (Supplementary Table S6). Finally, generalizability may be limited to Ontario, though similar disparities have been reported across Canada and internationally (Wanigaratne *et al.*, 2018; Zelkowitz *et al.*, 2015).

## Conclusion

Immigrant women with infertility in Ontario, particularly refugees, had slightly lower live birth rates, and fecundability than Canadian-born women. Addressing these disparities may require additional supports such as sensitive navigation, refugee-focused services, and integrated mental health care.

## Supplementary Material

deag144_Supplementary_Table_S1

deag144_Supplementary_Table_S2

deag144_Supplementary_Table_S3

deag144_Supplementary_Table_S4

deag144_Supplementary_Table_S5

deag144_Supplementary_Table_S6

deag144_Supplementary_Table_S7

deag144_Supplementary_Table_S8

deag144_Supplementary_Table_S9

deag144_Supplementary_Table_S10

deag144_Supplementary_Table_S11

## Data Availability

The dataset from this study is held securely in coded form at ICES. Although data-sharing agreements prohibit ICES from making the dataset publicly available, access may be granted to those who meet pre-specified criteria for confidential access, available at www.ices.on.ca/DAS. The full dataset, creation plan, and underlying analytic code are available from the authors upon request, with the understanding that the computer programs may rely upon coding templates or macros that are unique to ICES and are therefore either inaccessible or may require modification.
