# Supplementary material for: Reproductive outcomes among Canadian-born and immigrant women with infertility in Ontario: a population-based cohort study
Source: Hum Reprod. 2026 Sep 24;41(11):1993–2002. doi: 10.1093/humrep/deag144 (PMC13726753; doi:10.1093/humrep/deag144)
Supplement: Supplementary file 1 [file deag144_supplementary_table_s1.pdf]

Human Reproduction, 2026, 1–1
https://doi.org/10.1093/humrep/deag144
Supplementary Data

human reproduction

OXFORD

Supplementary Table S1. Countries included in each world region of origin among immigrant women in Ontario, 2010–2019.

World region	Countries included
Western Nations and Europe	Albania, Andorra, Australia, Austria, Belarus, Belgium, Bermuda, Bosnia and Herzegovina, Bulgaria, Croatia, Czech Republic, Denmark, Estonia, Finland, Former Czechoslovakia, Former USSR, Former Yugoslavia, Falkland Islands, France, Germany, Greece, Greenland, Hungary, Iceland, Ireland, Italy, Kosovo, Latvia, Liechtenstein, Lithuania, Luxembourg, Macedonia, Malta, Moldova, Monaco, Montenegro, Netherlands, New Zealand, Norway, Pitcairn Island, Poland, Portugal, Romania, Russian Federation, San Marino, Serbia, Serbia and Montenegro, Slovakia, Slovenia, Southern Antarctic Territories, Spain, St Helena, St Pierre and Miquelon, Sweden, Switzerland, Ukraine, the UK, the USA, Vatican City State.
Hispanic America	Argentina, Bolivia, Brazil, Chile, Colombia, Costa Rica, Cuba, Dominican Republic, Ecuador, El Salvador, French Guiana, Guatemala, Guyana, Honduras, Mexico, Nicaragua, Panama, Paraguay, Peru, Puerto Rico, Suriname, Uruguay, Venezuela.
Caribbean	Anguilla, Antigua and Barbuda, Aruba, Bahamas, Barbados, Belize, Cayman Islands, Dominica, Grenada, Guadeloupe, Haiti, Jamaica, Martinique, Montserrat, Netherlands Antilles, Saint Kitts and Nevis, Saint Lucia, Saint Vincent and the Grenadines, Trinidad and Tobago, Turks and Caicos Islands, Virgin Islands, British Virgin Islands, West Indies NES.
Sub-Saharan Africa	Angola, Benin, Botswana, Burkina Faso, Burundi, Cameroon, Cape Verde, Central African Republic, Chad, Comoros, Congo, Democratic Republic of Congo, Côte d'Ivoire, Djibouti, Equatorial Guinea, Eritrea, Ethiopia, Gabon, Gambia, Ghana, Guinea, Guinea-Bissau, Kenya, Lesotho, Liberia, Madagascar, Malawi, Mali, Mauritania, Mauritius, Mayotte, Mozambique, Namibia, Niger, Nigeria, Réunion, Rwanda, Sao Tome and Principe, Senegal, Seychelles, Sierra Leone, Somalia, South Africa, Swaziland, Tanzania, Togo, Uganda, Western Sahara, Zambia, Zimbabwe.
Middle East and North Africa	Algeria, Armenia, Azerbaijan, Bahrain, Cyprus, Egypt, Georgia, Iran, Iraq, Israel, Jordan, Kazakhstan, Kuwait, Kyrgyzstan, Lebanon, Libya, Morocco, Oman, Occupied Palestinian Territory, Qatar, Saudi Arabia, Sudan, Syria, Tajikistan, Tunisia, Turkey, Turkmenistan, United Arab Emirates, Uzbekistan, Yemen.
East Asia and Pacific	Asia NES, Brunei Darussalam, Cambodia, China, Fiji, French Polynesia, Guam, Hong Kong, Indonesia, Japan, Kiribati, North Korea, South Korea, Laos, Macau, Malaysia, Mongolia, Myanmar, Nauru, New Caledonia, Northern Mariana Islands, Palau, Papua New Guinea, Philippines, Samoa, Singapore, Solomon Islands, Taiwan, Thailand, Tibet, Tonga, Vanuatu, Vietnam, Tuvalu, Marinas, Marshall Islands, Micronesia (Federated States of), Cook Islands, Wallis and Futuna, East Timor, Oceania NES.
South Asia	Afghanistan, Bangladesh, Bhutan, India, Maldives, Nepal, Pakistan, Sikkim, Sri Lanka.
