# Supplementary material for: Reproductive outcomes among Canadian-born and immigrant women with infertility in Ontario: a population-based cohort study
Source: Hum Reprod. 2026 Sep 24;41(11):1993–2002. doi: 10.1093/humrep/deag144 (PMC13726753; doi:10.1093/humrep/deag144)
Supplement: Supplementary file 2 [file deag144_supplementary_table_s10.pdf]

Human Reproduction, 2026, 1–1
https://doi.org/10.1093/humrep/deag144
Supplementary Data

human reproduction

OXFORD

Supplementary Table S10. World Region of Origin among immigrant individuals with diagnosed infertility in Ontario, 2010–2019, by immigration category (N = 77 174).[§,‡]

World region of origin	Non-refugee immigrant (N = 66 131)	Refugee immigrant (N = 11 043)
Middle East and North Africa	16674 (25.2)	2031 (18.4)
East Asia and Pacific	9833 (14.9)	1007 (9.1)
South Asia	8492 (12.8)	802 (7.3)
Western Nations and Europe	5209 (7.9)	463 (4.2)
Hispanic America	2082 (3.1)	704 (6.4)
Caribbean	1621 (2.5)	280 (2.5)
Sub-Saharan Africa	1881 (2.8)	1665 (15.1)
Unknown/Missing	20339 (30.8)	20339 (30.8)

[§] World region derived from IRCC country of birth or citizenship data.
[‡] See Supplementary Table S5 for region classification definitions.
Note: Percentages calculated within immigration category.
