# Supplementary material for: Reproductive outcomes among Canadian-born and immigrant women with infertility in Ontario: a population-based cohort study
Source: Hum Reprod. 2026 Sep 24;41(11):1993–2002. doi: 10.1093/humrep/deag144 (PMC13726753; doi:10.1093/humrep/deag144)
Supplement: Supplementary file 3 [file deag144_supplementary_table_s11.pdf]

Human Reproduction, 2026, 1–1
https://doi.org/10.1093/humrep/deag144
Supplementary Data

human reproduction

OXFORD

Supplementary Table S11. Benjamini–Hochberg false discovery rate correction for secondary and exploratory analyses, Ontario, 2010–2019.

Analysis	Comparison	Raw *P*-value	FDR-adjusted *P*-value*	FDR interpretation
Main assisted conception	Non-refugee vs Canadian-born	<0.0001	0.00013	Significant
Main assisted conception	Refugee vs Canadian-born	<0.0001	0.00013	Significant
Main TTP/fecundability†	Non-refugee vs Canadian-born	<0.0001	0.00013	Significant
Main TTP/fecundability†	Refugee vs Canadian-born	<0.0001	0.00013	Significant
Pre-OFP live birth‡	Non-refugee vs Canadian-born	<0.0001	0.00013	Significant
Pre-OFP live birth‡	Refugee vs Canadian-born	<0.0001	0.00013	Significant
Post-OFP live birth‡	Non-refugee vs Canadian-born	0.1619	0.1619	Not significant
Post-OFP live birth‡	Refugee vs Canadian-born	0.0033	0.0039	Significant
Pre-OFP TTP/ fecundability†,‡	Non-refugee vs Canadian-born	<0.0001	0.00013	Significant
Pre-OFP TTP/ fecundability†,‡	Refugee vs Canadian-born	<0.0001	0.00013	Significant
Post-OFP TTP/ fecundability†,‡	Non-refugee vs Canadian-born	0.1588	0.1619	Not significant
Post-OFP TTP/ fecundability†,‡	Refugee vs Canadian-born	<0.0001	0.00013	Significant
Pre-OFP assisted conception‡	Non-refugee vs Canadian-born	<0.0001	0.00013	Significant
Pre-OFP assisted conception‡	Refugee vs Canadian-born	<0.0001	0.00013	Significant
Post-OFP assisted conception‡	Non-refugee vs Canadian-born	<0.0001	0.00013	Significant
Post-OFP assisted conception‡	Refugee vs Canadian-born	<0.0001	0.00013	Significant
YSL live birth§	<5 vs ≥5 years since landing	<0.0001	0.00013	Significant
YSL TTP/fecundability†,§	<5 vs ≥5 years since landing	<0.0001	0.00013	Significant
YSL assisted conception§	<5 vs ≥5 years since landing	0.0219	0.0245	Significant

Raw *P*-values and false discovery rate (FDR)-adjusted *P*-values are shown for 19 secondary and exploratory comparisons. Significance after FDR correction was assessed at $q < 0.05$.

* FDR-adjusted *P* values were calculated using the Benjamini–Hochberg procedure across 19 comparisons. Raw *P* values reported as <0.0001 were conservatively treated as 0.0001 for the FDR calculation.

† TTP = time to pregnancy; modelled using fecundability odds ratios.

‡ OFP = Ontario Fertility Program; pre-OFP = 2010–2014 and post-OFP = 2015–2019.

§ YSL = years since landing; analyses were conducted among immigrants and compared individuals with <5 vs ≥5 years since landing at index infertility diagnosis.

FDR, false discovery rate; OFP, Ontario Fertility Program; TTP, time to pregnancy; YSL, years since landing.

© The Author(s) 2026. Published by Oxford University Press on behalf of European Society of Human Reproduction and Embryology.
This is an Open Access article distributed under the terms of the Creative Commons Attribution License (https://creativecommons.org/licenses/by/4.0/), which permits unrestricted reuse, distribution, and reproduction in any medium, provided the original work is properly cited.
Human Reproduction, 2026, 00, 1–21
https://doi.org/10.1093/humrep/deag144
Original Article
