# Supplementary material for: Reproductive outcomes among Canadian-born and immigrant women with infertility in Ontario: a population-based cohort study
Source: Hum Reprod. 2026 Sep 24;41(11):1993–2002. doi: 10.1093/humrep/deag144 (PMC13726753; doi:10.1093/humrep/deag144)
Supplement: Supplementary file 4 [file deag144_supplementary_table_s2.pdf]

Human Reproduction, 2026, 1–1
https://doi.org/10.1093/humrep/deag144
Supplementary Data

human reproduction
OXFORD

Supplementary Table S2. Data sources accessed at ICES.

Dataset	Description
Ontario Health Insurance Plan (OHIP) Claims Database	Physician billing claims for inpatient and outpatient visits, consultations, and procedures. Used for infertility diagnosis (OHIP 628), mental health, and chronic conditions.
Canadian Institute for Health Information—Discharge Abstract Database (CIHI-DAD)	Hospital discharges, including demographic and clinical data. Used for obstetric outcomes, chronic, and mental health conditions.
Canadian Institute for Health Information—Same Day Surgery (CIHI-SDS)	Day surgery abstracts, including gynecologic procedures. Used for endometriosis procedures.
National Ambulatory Care Reporting System (NACRS)	Emergency and ambulatory care visits. Used for ED visits for mental health, obstetric outcomes.
Ontario Mental Health Reporting System (OMHRS)	Inpatient psychiatric admissions. Used for severe mental health conditions.
Registered Persons Database (RPDB)	Demographics, date of birth, sex, address, health card eligibility, and death. Used for age, censoring, Ontario residency.
Office of the Registrar General—Deaths (ORGD)	Vital statistics mortality file. Used for censoring at death.
Immigration, Refugees and Citizenship Canada (IRCC) Permanent Resident Database	Immigration data including landing date, immigration class, country of birth/citizenship. Used to define immigrant category and world region.
Postal Code Conversion File (PCCF)/Census/ONMARG	Geographic coding for neighbourhood income quintile, rurality, and marginalization.
Better Outcomes Registry & Network (BORN) Ontario	Maternal, perinatal, and fertility registry. Used for conception type, BMI, gestational age, prenatal visits, ultrasound, delivery outcomes.
NIDAY	Legacy perinatal database (2006–2012). Used for historical validation of deliveries.
MOMBABY Linked Dataset	Links maternal and newborn records in CIHI-DAD. Used for gestational age and delivery outcomes.
ICES Physician Database (IPDB)	Specialty of diagnosing physician. Used for infertility doctor type.
Derived ICES cohorts	ASTHMA, COPD, CHF, HIV, ODD (diabetes), HYPER (hypertension). Used for chronic condition flags.
