# Supplementary material for: Reproductive outcomes among Canadian-born and immigrant women with infertility in Ontario: a population-based cohort study
Source: Hum Reprod. 2026 Sep 24;41(11):1993–2002. doi: 10.1093/humrep/deag144 (PMC13726753; doi:10.1093/humrep/deag144)
Supplement: Supplementary file 5 [file deag144_supplementary_table_s3.pdf]

Human Reproduction, 2026, 1–1
https://doi.org/10.1093/humrep/deag144
Supplementary Data

human reproduction

OXFORD

Supplementary Table S3. Variables used to define cohort entry, exclusion criteria, and study exposures.

Assessment timing	Condition/criterion	Applicable codes	Data sources
Index event	Cohort entry	≥1 OHIP claim with infertility code 628 between 1 January 2010 and 31 December 2021	OHIP
At baseline	Age restriction	Exclude if age <18 or >55 years at index	RPDB
At baseline	Sex restriction	Exclude if male or missing sex	RPDB
At baseline	Residency	Exclude non-Ontario residents at index (substr(prcddablk , 1,2) ≠ 35)	RPDB
Lookback (2 years)	OHIP eligibility	Exclude if <2 years of OHIP eligibility before index	CONTACT/RPDB
Lookback (2 years)	Prior infertility	Exclude if any OHIP 628 billing in lookback window	OHIP
At baseline	Death before index	Exclude if death date < index date	RPDB/ORGD
At baseline	Duplicates	Retain earliest record per IKN; remove duplicates	Derived
At baseline	Immigration status	Exclude if immigration category unknown/other	IRCC
At baseline	Index year restriction	Exclude index year ≥2020 to ensure 2-year follow-up	OHIP
Exposure	Immigration category (imm_cat)	0 = Canadian-born; 1 = Non-refugee immigrant; 2 = Refugee immigrant	IRCC
