# Supplementary material for: Reproductive outcomes among Canadian-born and immigrant women with infertility in Ontario: a population-based cohort study
Source: Hum Reprod. 2026 Sep 24;41(11):1993–2002. doi: 10.1093/humrep/deag144 (PMC13726753; doi:10.1093/humrep/deag144)
Supplement: Supplementary file 6 [file deag144_supplementary_table_s4.pdf]

Human Reproduction, 2026, 1–1
https://doi.org/10.1093/humrep/deag144
Supplementary Data

human reproduction OXFORD

Supplementary Table S4. Variables used to define study outcome.

Outcome	Definition/codes	Data sources
Live birth (≥23 weeks)	Delivery with PREGNANCY_OUTCOME_ID = 1 021 030 and GA ≥23 weeks; exclude if NEODEATH = 2 (stillbirth)	BORN, NIDAY, MOMBABY
Time to pregnancy	Index consultation date → estimated conception (delivery date—gestational age). Censor at 2 years, loss of OHIP eligibility, or death.	BORN (B_BDATE, GA_AT_BIRTH_WEEKS), MOMBABY; RPDB for censoring
Conception type	IVF/ICSI = 1 013 130, 1 013 140 (NIDAY = 3,4); OI/IUI = 1 013 110, 1 013 120, 1 013 150, 3 000 006 (NIDAY = 2,5); Spontaneous = 1 013 160 (NIDAY = 1); Unknown = 1 013 180 (NIDAY = 0,9)	BORN (CONCEPTION_TYPE_ID), NIDAY (REPASS)
