# Supplementary material for: Reproductive outcomes among Canadian-born and immigrant women with infertility in Ontario: a population-based cohort study
Source: Hum Reprod. 2026 Sep 24;41(11):1993–2002. doi: 10.1093/humrep/deag144 (PMC13726753; doi:10.1093/humrep/deag144)
Supplement: Supplementary file 7 [file deag144_supplementary_table_s5.pdf]

Human Reproduction, 2026, 1–1
https://doi.org/10.1093/humrep/deag144
Supplementary Data

human reproduction

OXFORD

Supplementary Table S5. Variables used to define covariates.

Assessment timing	Condition/covariate	Applicable codes	Data sources
At index	Maternal age	Continuous, from RPDB BDATE	RPDB
At index	Parity	Parous if prior delivery in MOMBABY; Nulliparous if none	MOMBABY
At index	Rurality	Urban = RIO <40; Rural = RIO ≥40	PCCF
At index	Income quintile	Derived from postal code, census linkage (Q1–Q5)	RPDB + Census
At index	Immigration category	0 = Canadian-born; 1 = Non-refugee; 2 = Refugee	IRCC
At index	World region of origin	From COUNTRY_OF_BIRTH or fallback to citizenship; grouped by CIC world area format	IRCC
At index	Years since landing	Continuous; categorical: <5, 5–9, ≥10, temporary resident	IRCC (LANDING_DATE)
At index	Infertility physician specialty	OB/GYN = 20; Family physician = 00; Other = all else	OHIP/IPDB
At index and 2-year lookback	Mental health composite	ICD-10: F10–F19, F55 (substance); F20 etc. (psychotic); F30–39, F40–48, F93.1, F93.2 (mood/anxiety); deliberate self-harm codes; OHIP psychiatrist/FP outpatient algorithm. Requires ≥1 inpatient/ED OR ≥2 outpatient	OHIP, DAD, NACRS, OMHRS
At index and 2-year lookback	Chronic health composite	HIV (ICES HIV), Hypertension (HYPER), Diabetes (ODD), Asthma (ASTHMA), CHF (CHF), COPD (COPD), Autoimmune (ICD-10 M32), Endocrine (E03.9, E07.9, E23.7, E25.0, E27.1–E27.4), Thrombosis (O22.5, O87.3, D68.69)	ICES derived cohorts, DAD, OHIP
At pregnancy or 2-year lookback	Obesity	BORN BMI ≥30; OHIP dx 278; ICD-10 E6601, E6609, E661, E668, E669	BORN, OHIP, DAD
At index	PCOS	OHIP dx 256 OR (hirsutism 709 + irregular menses 626); any time frame	OHIP
At index	Endometriosis	CIHI-DAD: N80; OHIP: 2× code 617 with suffix A; SDS: procedure + N80	OHIP, DAD, SDS
