# Supplementary material for: Reproductive outcomes among Canadian-born and immigrant women with infertility in Ontario: a population-based cohort study
Source: Hum Reprod. 2026 Sep 24;41(11):1993–2002. doi: 10.1093/humrep/deag144 (PMC13726753; doi:10.1093/humrep/deag144)
Supplement: Supplementary file 8 [file deag144_supplementary_table_s6.pdf]

Human Reproduction, 2026, 1–1
https://doi.org/10.1093/humrep/deag144
Supplementary Data

human reproduction

OXFORD

Supplementary Table S6. Association between immigrant status and reproductive outcomes, Ontario, 2010–2019 (N = 254 463).

Outcome	Comparison	Mental health (MH) status	Adjusted RR/FOR* (95% CI)	*P*-value	Interaction *P*-value†
Live birth within 2 years	Non-refugee vs Canadian-born	No MH	0.87 (0.86–0.88)	<0.0001	0.30
		With MH	0.76 (0.73–0.79)	<0.0001	
	Refugee vs Canadian-born	No MH	0.87 (0.84–0.90)	<0.0001	
		With MH	0.77 (0.70–0.84)	<0.0001	
Assisted conception	Non-refugee vs Canadian-born	No MH	0.71 (0.69–0.75)	<0.0001	0.09
		With MH	0.69 (0.62–0.75)	<0.0001	
	Refugee vs Canadian-born	No MH	0.65 (0.60–0.70)	<0.0001	
		With MH	0.50 (0.40–0.63)	<0.0001	
Time to pregnancy (FOR)	Non-refugee vs Canadian-born	No MH	0.95 (0.94–0.96)	<0.0001	<0.001
		With MH	1.03 (0.99–1.07)	0.12	
	Refugee vs Canadian-born	No MH	0.87 (0.84–0.90)	<0.0001	
		With MH	0.95 (0.88–1.03)	0.22	

Adjusted relative risks (RRs) or fecundability odds ratios (FORs) estimated from log binomial or discrete-time Cox models including an interaction term between immigrant status and mental health condition. Canadian-born women without a mental health condition served as the reference group. *Data shown as frequency (percentage) with crude and adjusted relative risks (RR) or median (interquartile range) when indicated with fecundability odds ratios (FOR) and 95% CIs*.

* Adjusted for age, parity, income quintile, rurality, and chronic health conditions.

† *P*-value for interaction term (immigrant category × mental health) from likelihood ratio or Wald test.
