# Supplementary material for: Reproductive outcomes among Canadian-born and immigrant women with infertility in Ontario: a population-based cohort study
Source: Hum Reprod. 2026 Sep 24;41(11):1993–2002. doi: 10.1093/humrep/deag144 (PMC13726753; doi:10.1093/humrep/deag144)
Supplement: Supplementary file 9 [file deag144_supplementary_table_s7.pdf]

Human Reproduction, 2026, 1–1
https://doi.org/10.1093/humrep/deag144
Supplementary Data

human reproduction

OXFORD

Supplementary Table S7. Association between immigrant status and reproductive outcomes before and after implementation of the Ontario Fertility Program (OFP) in 2015, Ontario, 2010–2019 (N = 254 463).

Outcome	Era	Canadian-born	Non-refugee immigrant*	Refugee immigrant*
Live birth within 2 years	2010–2014	Ref	0.94 (0.93–0.96)	0.88 (0.85–0.92)
	2015–2019	Ref	0.99 (0.97–1.00)	0.95 (0.91–0.98)
Assisted conception‡	2010–2014	Ref	0.74 (0.71–0.77)	0.64 (0.58–0.71)
	2015–2019	Ref	0.71 (0.68–0.74)	0.64 (0.58–0.71)
Time to pregnancy†	2010–2014	Ref	0.96 (0.94–0.97)	0.87 (0.83–0.91)
	2015–2019	Ref	0.99 (0.99–1.01)	0.91 (0.88–0.96)

Non-refugee and refugee immigrant status each compared with Canadian-born. *Data shown as number with outcome/number at risk (%), adjusted relative risks (RR) or fecundability odds ratios (FOR) with 95% CIs*.

* Model adjusted for age, parity, income quintile, rurality, and chronic health conditions.

† Time to pregnancy calculated among those achieving pregnancy; FOR, fecundability odds ratio.

‡ Assisted conception analyses restricted to individuals with a pregnancy; includes IVF, IVF with ICSI, ovulation induction (OI), and IUI.
