# Supplementary material for: Reproductive outcomes among Canadian-born and immigrant women with infertility in Ontario: a population-based cohort study
Source: Hum Reprod. 2026 Sep 24;41(11):1993–2002. doi: 10.1093/humrep/deag144 (PMC13726753; doi:10.1093/humrep/deag144)
Supplement: Supplementary file 10 [file deag144_supplementary_table_s8.pdf]

Human Reproduction, 2026, 1–1
https://doi.org/10.1093/humrep/deag144
Supplementary Data

human reproduction
OXFORD

Supplementary Table S8. Association between years since landing and reproductive outcomes, Ontario, 2010–2019, N = 77 174.

Outcome	Exposure	n/N (%)	Crude RR/FOR (95% CI)	Adjusted* RR/FOR (95% CI)
Live birth within 2 years	≥5 years since landing	18504/57694 (32.1)	Reference	Reference
	<5 years since landing	7050/18402 (38.3%)	1.19 (1.17–1.22)	1.08 (1.05–1.10)
Assisted conception‡	≥5 years since landing	4226/24346 (17.4)	Reference	Reference
	<5 years since landing	1523/9169 (16.6%)	0.96 (0.91–1.01)	0.94 (0.89–0.99)
Time to pregnancy†	≥5 years since landing	11 (4–25), N = 24346	Reference	Reference
(median, IQR, months)	<5 years since landing	11 (4–24), N = 9169	1.27 (1.25–1.30)	1.09 (1.07–1.12)

Data shown as frequency (percentage) with crude and adjusted relative risks (RR) or median (interquartile range) when indicated with fecundability odds ratios (FOR) and 95% CIs.
* Model adjusted for age, parity, income quintile, rurality, and chronic health conditions.
† Time to pregnancy calculated among those achieving pregnancy; FOR, fecundability odds ratio.
‡ Assisted conception analyses restricted to individuals with a pregnancy; includes IVF, IVF with ICSI, ovulation induction (OI), and IUI.
Note: 1078 missing for pregnancy and live birth within 2 years and time to pregnancy, 472 missing from assisted conception among pregnancies.
