# Supplementary material for: Reproductive outcomes among Canadian-born and immigrant women with infertility in Ontario: a population-based cohort study
Source: Hum Reprod. 2026 Sep 24;41(11):1993–2002. doi: 10.1093/humrep/deag144 (PMC13726753; doi:10.1093/humrep/deag144)
Supplement: Supplementary file 11 [file deag144_supplementary_table_s9.pdf]

Human Reproduction, 2026, 1–1
https://doi.org/10.1093/humrep/deag144
Supplementary Data

human reproduction
OXFORD

Supplementary Table S9. Comparison of primary and sensitivity analyses after excluding recent immigrants, Ontario, 2010–2019.

Outcome	Comparison	Primary adjusted RR/FOR (95% CI)*	Sensitivity adjusted RR/FOR (95% CI)†	Change in estimate‡
Live birth within 2 years	Non-refugee immigrants vs Canadian-born	0.97 (0.95–0.98)	0.94 (0.93–0.95)	−0.03
Live birth within 2 years	Refugee immigrants vs Canadian-born	0.91 (0.89–0.94)	0.92 (0.89–0.95)	+0.01
Time to pregnancy/ fecundability§	Non-refugee immigrants vs Canadian-born	0.97 (0.96–0.99)	0.94 (0.93–0.96)	−0.03
Time to pregnancy/ fecundability§	Refugee immigrants vs Canadian-born	0.89 (0.86–0.91)	0.90 (0.87–0.93)	+0.01
Assisted conception¶	Non-refugee immigrants vs Canadian-born	0.72 (0.70–0.75)	0.73 (0.71–0.76)	+0.01
Assisted conception¶	Refugee immigrants vs Canadian-born	0.64 (0.60–0.69)	0.68 (0.63–0.73)	+0.04

Data are shown as adjusted relative risks (RRs) or fecundability odds ratios (FORs) with 95% CIs. The sensitivity analysis excluded immigrants who had arrived in Canada within 5 years of the index infertility diagnosis, while retaining Canadian-born women as the reference group.

* Primary models used the full analytic cohort and compared non-refugee and refugee immigrants with Canadian-born women.

† Sensitivity models excluded immigrants with <5 years since landing at the index infertility diagnosis; Canadian-born women were retained as the reference group.

‡ Change in estimate calculated as sensitivity estimate minus primary estimate.

§ Time to pregnancy was modelled using fecundability odds ratios (FORs). FOR <1 indicates lower fecundability, corresponding to a longer time to pregnancy.

¶ Assisted conception analyses were restricted to individuals who achieved a pregnancy and include IVF, ICSI, ovulation induction (OI), and IUI.

All models were adjusted for age, parity, neighbourhood income quintile, rurality, and chronic health conditions.
